# Evaluation of Functionalized Birch Bark Extracts in Epoxy Thermosets

**DOI:** 10.1021/acs.biomac.6c00228

**Published:** 2026-06-24

**Authors:** Heather M. LaFrance, John D. Chea, Emre Kinaci, Kylie D. Howard, James A. Newell, Joseph F. Stanzione

**Affiliations:** † Department of Chemical Engineering, 3536 Rowan University, 201 Mullica Hill Rd., Glassboro, New Jersey 08028, United States; ‡ Advanced Materials and Manufacturing Institute (AMMI), 3536 Rowan University, 107 Gilbreth Pkwy, Mullica Hill, Glassboro, New Jersey 08062, United States

## Abstract

Birch bark, often discarded or burned as a low-grade fuel in the pulp and paper industry, contains valuable compounds with significant potential as biobased polymer precursors. Among these are triterpenoids such as betulin, betulinic acid, and lupeol, which comprise roughly 10–40% of the dried bark mass, alongside lignocellulosic and suberin fractions. In this work, crude birch bark extract (BBE) was extracted and used directly as a polymer precursor material. Epoxidized BBE was cured with a range of biobased and petroleum-derived amines, including furan-derived difuran diamine (DFDA). The resulting thermoset polymers demonstrated high extents of cure (greater than 99%) and room-temperature storage moduli, glass transition temperatures, and thermal stabilities comparable to those of petroleum-derived epoxy systems.

## Graphical abstract

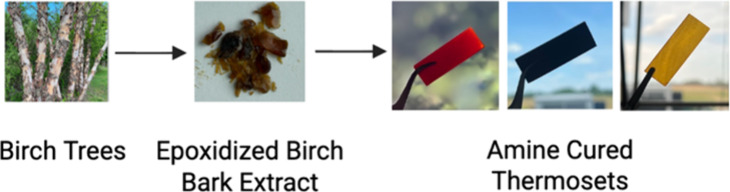

## 1 Introduction

The demand for sustainable alternatives to petroleum-derived plastics continues to grow in tandem with increased fossil fuel use and associated negative environmental impacts.^1^ Currently, 4–8% of the annual global oil consumption is attributed to plastic manufacturing, with production volumes continually increasing to meet demand; therefore, the development and design of renewable feedstocks are essential for reducing reliance on fossil resources.^2^ Biobased polymers are a topic of study to replace traditional plastics, but the commercial implementation remains slow due to cost, processing efficiency, and end-of-life considerations.^3–9^

Wood, bark, and grasses provide an attractive path forward for their valuable raw materials and lack of competition with food supplies.^10,11^ These nonfood sources are produced in large quantities by forestry and pulp and paper industries, up to 40 tons of birch bark per site daily; however, this low-grade fuel is valued at $5–7 per ton or simply discarded.^12–14^ The combustion of birch barks for fuel eliminates the opportunities to utilize betulin, betulinic acid, lupeol, and other birch extract components to their fullest potential.

The triterpenoid components of birch bark (betulin, betulinic acid, and lupeol), shown in Figure 1, have historically been used in various medical applications, including but not limited to, as the main ingredient in antioxidant, anticancer, anti-HIV, anti-inflammatory, antimicrobial, antifungal, antipigmentation, and antiseptic formulations.^12,13,15–17^ The extracts of all birch species typically contain pentacyclic triterpenoids, lupanes, and oleananes; as such, the birch bark triterpenoid content is typically reported as being between 10 and 40% on a dry mass basis, with most of the triterpenoid mass comprising betulin, betulinic acid, and lupeol.^13,14,18^ Other substances such as betulinic aldehyde, oleanolic acid, erithrodiol, and betulin-3-caffeate are present in much lower quantities. Suberin accounts for up to 45% of birch bark on a dry mass basis, while the remainder contains polysaccharides (6%) and lignin (9%).^14^ Suberin is a natural hydrophobic biopolyester responsible for forming a protective barrier in plant tissues and controlling water transport; a representative structure is shown in the Supporting Information (SI) (see Figure S1).^19,20^ Although each component contributes to the overall composition of birch bark extract (BBE), it complicates the selective isolation of individual triterpenoids, such as betulin.^22^

**Figure 1:**
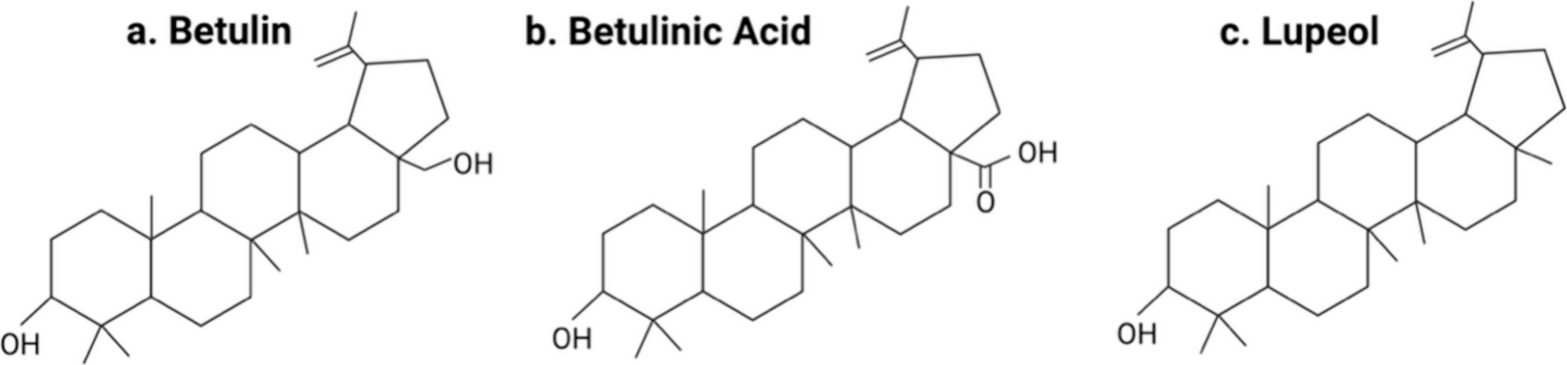
Chemical structures of the primary triterpenoid components of BBE: (a) betulin, (b) betulinic acid, and (c) lupeol.

The commercialization of betulin-based polymers has been limited by the high cost and complexity of purification required to isolate natural products. Isolating pure betulin requires multiple extraction and recrystallization steps, which often outweigh the benefits of isolating and purifying the chemical itself.^18^ Furthermore, most studies have focused on purified compounds rather than crude BBE, with room left for researchers to answer the question whether extracts themselves could serve as a practical renewable feedstock for the development of viable biobased polymers.^18,21,22^

This work investigates the viability of functionalizing minimally processed BBE as a renewable feedstock for the synthesis of biobased epoxy resins. BBE was directly epoxidized to determine whether the natural mixture of triterpenoids and hydroxyl-containing compounds could produce functional epoxy resins. The epoxidized birch bark extract (EBBE) was blended with petroleum-based epoxy resins and cured with different amines to study how resin composition and curing agent affect thermomechanical properties. To the best of our knowledge, this is the first report showing that BBE, rather than pure betulin, can be directly incorporated into epoxy thermosets. This approach offers a more practical route for developing biobased thermoset epoxy polymers by lowering processing costs, reducing reliance on petroleum feedstocks, and making use of an underutilized natural resource.

## 2 Experimental Methods

### 2.1 Materials

Birch bark (Betula nigra) was collected monthly from the Jean and Ric Edelman Fossil Park of Rowan University, NJ, USA (39.760801, −75.125030). Chloroform (99.8%), methanol HiPerSolv CHROMANORM (99.8%), acetonitrile (ACN) HiPerSolv CHROMANORM (99.9%), tetrahydrofuran (THF) HiPerSolv CHROMANORM (99.7%), sodium hydroxide (50% w/w), and dichloromethane (DCM) ACS grade were purchased from VWR Chemicals BDH. Ethanol (200 proof) was purchased from Pharmco Products Inc. Epichlorohydrin (EpCl) (99%) was purchased from Acros Organics. Tetrabutylammonium hydrogen sulfate (TBAHS) (97%) was purchased from Sigma-Aldrich. Magnesium sulfate anhydrous (99.5%), acetic anhydride, pyridine (99+%), and chloroform-d for NMR (99.8% D) were purchased from Thermo Fisher Scientific. Perchloric acid (0.1 N) was purchased from Alfa Aesar. Glacial acetic acid (100%) was purchased from SUPELCO. EPIKURE Curing Agent W (EPIKURE W) was purchased from Miller-Stephenson Chemical Company, INC. Cardolite NC-558 was obtained from Cardolite Corporation. 5,5′-Methylenedifurfurylamine (DFDA) was synthesized following the method reported by Hu et al.^23^

### 2.2 Extraction of Birch Bark

Birch bark was peeled monthly from river birch trees (Betula nigra) at the Jean and Ric Edelman Fossil Park of Rowan University (39.760801, −75.125030). An industrial lab-grade blender was used to process the bark before Soxhlet extraction. Approximately 600 mL of chloroform was utilized per 120 g of extract and heated under reflux for 24 h. Along with the chloroform extraction, ethanol was also evaluated as an extraction solvent using the exact same process and conditions. The final product was filtered to separate solid particles, and the extract solutions were dried using a rotary evaporator. Images of the dried chloroform and ethanol BBE are presented in the SI (see Figures S2 and S3).

### 2.3 Epoxidation of Birch Bark Extract (BBE)

EBBE was synthesized via an epoxidation reaction using a 30-molar excess of EpCl and using TBAHS as a phase-transfer catalyst at a 0.16 molar ratio relative to BBE. Betulin is shown as a representative component of BBE, in Scheme 1, to show the epoxidation reaction pathway. An excess amount of EpCl was needed to ensure sufficient contact with the betulin molecules in the mixture, pushing the reaction toward near-optimal conversion while also minimizing the potential for appreciable, undesired oligomerization.^24,25^ The reaction was stirred at 90 °C for 72 h under reflux, followed by a dropwise addition of 50% sodium hydroxide (NaOH) solution in water to convert chlorohydrin to the epoxide groups. The resultant product formed visible salts; therefore, DCM was used to extract the product, which was subsequently washed once with deionized water and four times with brine at a 1:1 volume ratio. The final organic phase was dried using anhydrous magnesium sulfate and then filtered and distilled to remove any excess DCM and EpCl. An image of the EBBE is presented in the SI (see Figure S4).

**Scheme 1:**
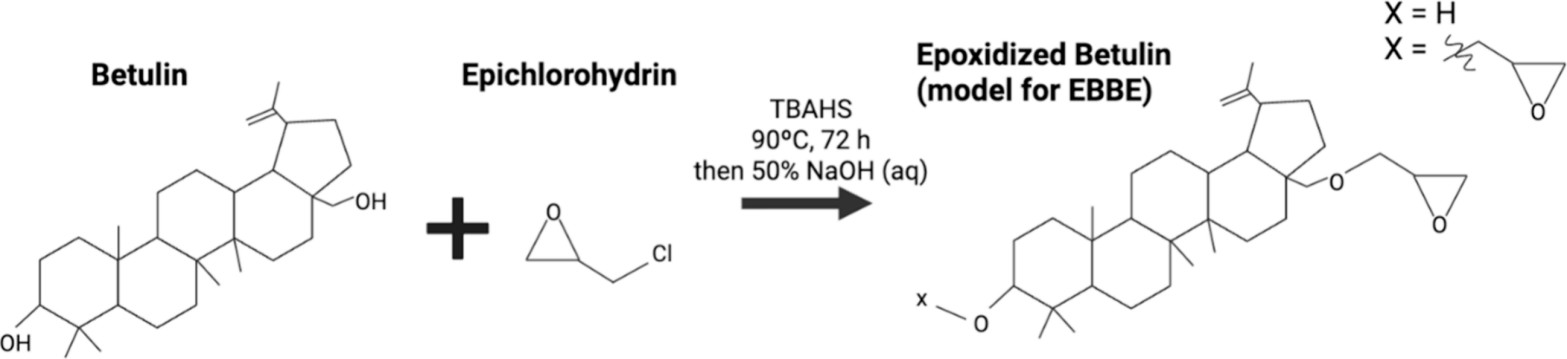
Epoxidation of Betulin Using Epichlorohydrin under Phase-Transfer Conditions^a^

a This was followed by the addition of sodium hydroxide to form epoxide groups, yielding epoxidized betulin as a model compound for EBBE.

### 2.4 Resin Blend

A comparison of a resin blend of EBBE with the diglycidyl ether of hydrogenated bisphenol A (Eponex 1510) was performed. Eponex 1510, a cyclo-aliphatic petroleum-derived commercial epoxy resin, was chosen as a control for property comparison because of its cyclo-aliphatic/aliphatic nature, similar to BBE epoxies.^26^ The percentage of EBBE introduced into the Eponex 1510 blend was increased up to 50% EBBE by weight. Any sample with higher than 50% EBBE exhibited brittleness; therefore, no further action occurred on such systems. As a solid epoxy, there is an inherent processing challenge with the EBBE blends that require mixing at a temperature above the melting point of the epoxy. The EBBE may be dissolved and blended with a liquid resin such as Eponex 1510 and still exhibit high mixture stability. The blends were cured with various amine hardeners, such as aromatic EPIKURE W, and biobased sources, such as Cardolite NC-558, a CNSL-derived phenalkamine 28, and DFDA, a furan-based diamine found in agricultural waste materials.^27^ Stoichiometric amounts of amine were used relative to the epoxy functionality, determined by epoxy equivalent weight (EEW) titrations, with no excess amine used. Therefore, near-optimal conversion was achieved through the applied temperature and curing time for each epoxy blend. All blended epoxy and amine mixtures were mixed and degassed via a Thinky ARE planetary mixer, cast into rubber molds, cured at 125 °C for 18 h, and then postcured at 150 °C for 3 h to ensure complete curing. Samples cured with EPIKURE W required a higher temperature for complete curing due to the long working life of the material. EPIKURE W is meant to be stable at room temperature for >20 h before hardening; therefore, a higher temperature (200 °C) and longer time (5 h) were used for the postcure.^28,29^

### 2.5 Structural Characterization

#### 2.5.1 High-Performance Liquid Chromatography (HPLC)

We employed a method based on a previous study by Maji et al.^30^ The mobile phase composition was 94% ACN with 0.1% acetic acid and 6% water at a 1 mL/min isocratic flow rate, and the Xterra MS C18, a reversed-phase silica-packed column, was chosen as the stationary phase.^30^ All samples were dissolved in HPLC-grade methanol at a 10 mg sample/50 mL solvent ratio and then analyzed at a column temperature of 25 °C over 20 min at a 210 nm wavelength. The resulting chromatograms were analyzed for the concentrations of betulin, betulinic acid, and lupeol, with the remaining peaks classified as unknown. The HPLC plot of the BBEs is provided in the Results section, Figure 2, and the SI (see Figures S5 and S6).

**Figure 2:**
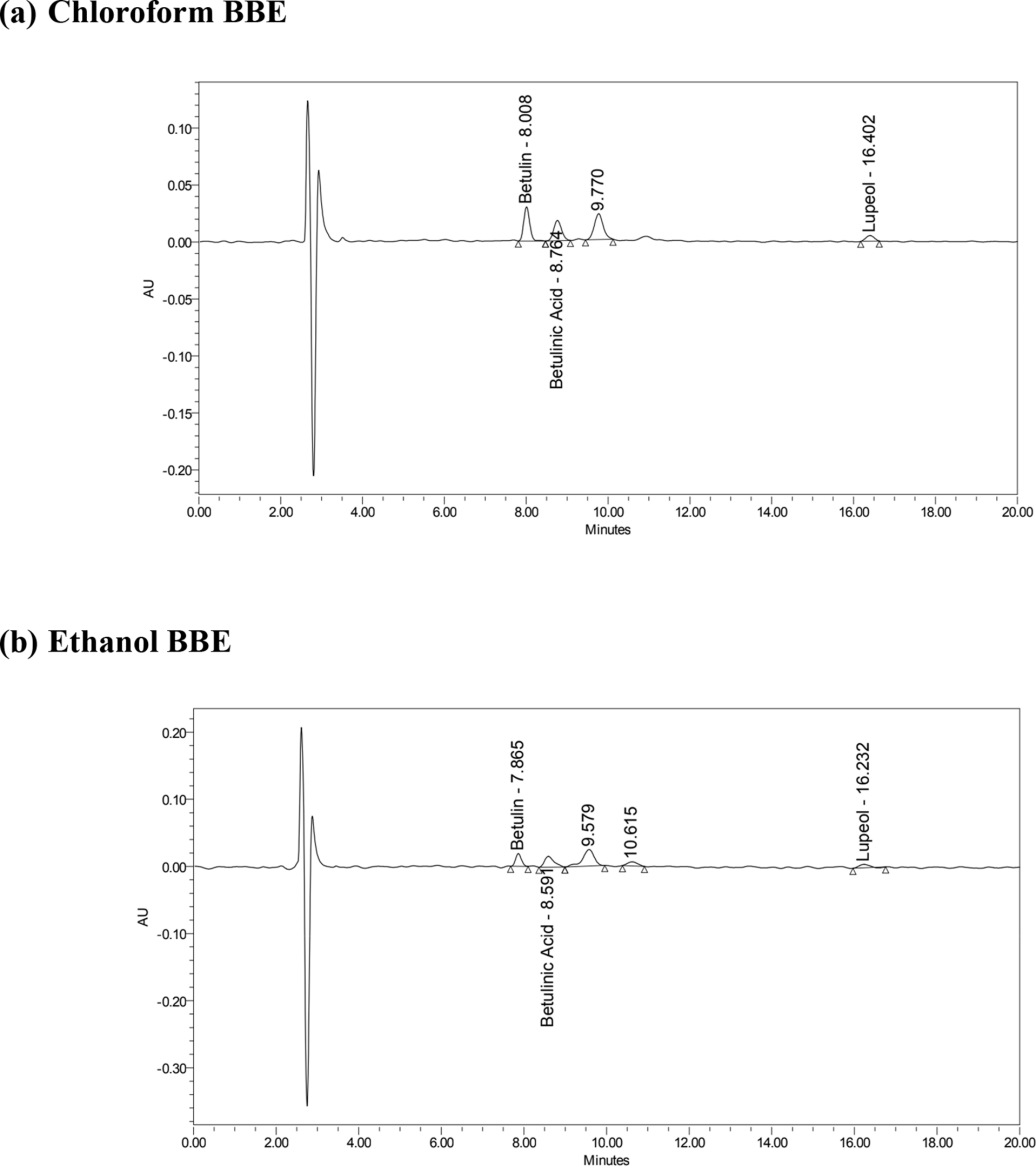
HPLC chromatograms of BBE obtained using (a) chloroform and (b) ethanol. Peaks corresponding to betulin, betulinic acid, and lupeol were identified based on retention times and comparison with standards.

#### 2.5.2 Advanced Polymer Chromatography

A Waters ACQUITY APC was used with a refractive index detector to determine the molecular weight distributions of the materials extracted and synthesized. The samples were run in THF at a flow rate of 0.6 mL/min using a series of ACQUITY APC columns (XT 450 2.5 μm, XT 125 μm, and XT 45 1.7 μm). APC plots of the BBE and epoxy are available in the SI (see Figures S7–S9).

#### 2.5.3 Nuclear Magnetic Resonance (NMR) Spectrometry

A Bruker Avance Core NMR spectrometer was used to obtain the ^1^H NMR spectra analysis of each extract and each synthesized monomer to confirm the chemical structure and key functional groups. Samples were dissolved in deuterated chloroform, and 16 scans were collected at 25 °C at 400 MHz. All NMR spectra are shown in the SI (see Figure S10).

#### 2.5.4 Hydroxyl Value Titration

Hydroxyl values of each extract were determined by acetic anhydride acetylation following ASTM E222–23.^31^ A known amount of extract was acetylated in a solution of acetic acid in pyridine under reflux for 1.5 h. After reflux, excess acetic anhydride was hydrolyzed with water to create acetic acid. The solution was then titrated with a 0.5 N sodium hydroxide solution, and the difference between the blank and sample titrations was used to calculate the hydroxyl content.

#### 2.5.5 Epoxy Equivalent Weight (EEW)

The EEW of each sample was determined through titrations using ASTM D1652.^32^ A known amount of the epoxy sample was dissolved in dichloromethane and reacted in a solution of tetraethylammonium bromide in glacial acetic acid with crystal violet indicator. The resulting solution was titrated with 0.1 N perchloric acid in glacial acetic acid. In this method, hydrogen bromide reacts stoichiometrically with epoxy groups to form bromohydrins, and the amount of acid consumed is used to quantify the epoxy content.

#### 2.5.6 Fourier Transform Infrared (FTIR) Spectroscopy

The conversion of epoxy and amine groups was confirmed using a Nicolet iS50 FTIR spectrometer in the near-infrared (NIR) region (4000–8000 cm^–1^). Before cure and after postcure, samples were examined at an 8 cm^–1^ resolution while collecting 32 scans per spectrum at ambient conditions. The peak at 4530 cm^–1^, which corresponds to vibrations related to oxirane groups, was tracked along with peaks corresponding to vibrations related to amines, with primary amines at 5900 cm^–1^ and primary and secondary amines at 6600 cm^–1^. Equation 1 was used to calculate the extent of cure. In eq 1, *A*(0) represents the reduced absorbance of the relevant IR peak before cure and *A*(*t*) represents the one after postcure. The FTIR plots are available in the SI (see Figures S11–S19).

$$\alpha =1-\frac{A\left(t\right)}{A\left(0\right)}$$ 1

### 2.6 Polymer Characterization

#### 2.6.1 Thermogravimetric Analysis (TGA)

The thermal stability of the epoxy thermosets was measured using a TA Instruments Discovery Series TGA 550. The samples were placed in aluminum pans and equilibrated to 30 °C, followed by heating to 700 °C at the rate of 10 °C/min under an inert (N_2_) atmosphere. Samples were run in triplicate.

#### 2.6.2 Differential Scanning Calorimetry (DSC)

Any primary and secondary transitions within the thermosets as a function of temperature were observed using a TA Instruments Discovery Series DSC 2500. 10–15 mg of cured sample was placed into a hermetic aluminum DSC pan and thermally scanned from −50 to 200 °C under N_2_. Samples were run in triplicate. The DSC plots are all provided in the SI (see Figures S20–22).

#### 2.6.3 Gel Content of Cured Samples

The gel content of each sample was determined by Soxhlet extraction in toluene for 24 h.^33^ A preweighed, dried sample was placed in an extraction thimble and extracted under reflux. After extraction, the insoluble fraction was dried to constant mass and used to calculate the gel content, as shown in eq 2.

$$\mathrm{Gel}\%=\frac{W_{\mathrm{final}}}{W_{\mathrm{initial}}}\times 100$$ 2

#### 2.6.4 Dynamic Mechanical Analysis (DMA)

DMA was performed using a TA Instruments Discovery Series DMA 850 on rectangular specimens with approximate dimensions of 17.5 × 12.5 × 3.0 mm to verify the glass transition temperature (*T*_g_) and elastic moduli of the postcured thermosets. All samples were thermally scanned from −50 °C to well above their *T*_g_ values at a frequency of 1 Hz, an amplitude of 10 μm, and a heating rate of 2 °C/min. Samples were run in triplicate. *T*_g_ values of the cured resins were obtained from the peak positions of both loss modulus (*E*″) and tan δ curves. The cross-link densities (*v*) were also obtained from storage modulus curves at 60 °C above the loss modulus *T*_g_ according to the theory of rubber elasticity using eq 3, where *R* is the ideal gas constant and *T* is the absolute temperature. The DMA plots are all provided in the SI (see Figures S20–22).

$$v=\frac{E^{\prime }}{3RT}$$ 3

## 3 Results and Discussion

BBEs were obtained using chloroform and ethanol, and both extraction yields generally ranged between 10 and 15% by mass. The molecular weight distribution and the weight percentages of betulin, betulinic acid, and lupeol were determined using APC and HPLC, respectively. As shown in Table 1, the extraction solvent significantly influences the composition of the resulting extracts. Chloroform BBE exhibited a higher total triterpenoid content, 37.2%, compared to ethanol BBE, 27.6%. Ethanol BBE contained a higher amount of nontriterpenoid components (other), 72.4%, compared to chloroform, 62.8%. The identities of the other % have been determined in the literature, and they include suberin, betulinic aldehyde, methyl betulinate, lupenone, aromatics, and lignin, with the majority being suberin.^12,21^ The molecular weight of the extract was used to determine its hydroxyl values and estimate the number of reactive groups present for functionalization, which were found to be comparable between solvents.

**Table 1:** Birch Bark Extract Composition Using Chloroform and Ethanol as Extractive Solvents^a^

**birch bark extracts** (BBEs)	**betulin (%)**	**betulinic acid (%)**	**lupeol (%)**	**total (%)**	**other (%)**	***M***_**n**_ (g/mol)	**hydroxyl value** (mg OH/g sample)
chloroform	13.2 ± 1.6	17.2 ± 1.2	6.77 ± 0.5	37.2 ± 3.3	62.8 ± 3.3	501 ± 10	126 ± 11
ethanol	9.35 ± 2.5	12.5 ± 1.6	5.73 ± 0.9	27.6 ± 5.0	72.4 ± 5.0	578 ± 15	159 ± 26

a All samples shown were extracted from river birch trees found at the Jean and Ric Edelman Fossil Park of Rowan University.

Representative HPLC chromatograms of BBE, as shown in Figure 2, confirm the presence of the major triterpenoids betulin, betulinic acid, and lupeol. Triterpenoids are the major reactive components of BBE and possess either hydroxyl groups or carboxylic acid groups that can be functionalized. In this case, betulin, having two hydroxyl groups, is predicted to act as the major reactive component, while betulinic acid and lupeol, having one hydroxyl group each, might not contribute to the reaction. It is important to note that the solvent used for extraction affects the quantity of triterpenoid compounds present in the extract, along with the number of reactive functional groups. On the basis of the higher triterpenoid content and availability of reactive hydroxyl groups, chloroform BBE samples were chosen for further functionalization studies.

Chloroform BBE was used as the feedstock for epoxidation to produce EBBE, as described in the Experimental Section. The resulting EBBE material appeared as a solid, amber-colored resin with semitransparent and homogeneous characteristics. After synthesizing the EBBE, the EEWs of the Eponex 1510 and EBBE products were determined and are shown in Table 2 (see Figures S7–S9 for APC data). The EEW for EBBE indicated that one epoxy group was present per molecule, on average. The epoxidation of BBE resulted in one glycidyl group per molecule, on average, due to the triterpenoids, mainly betulin, containing only one primary alcohol, which is significantly more reactive toward glycidation, via our method of epoxidation, than secondary alcohols, although such reactions may occur. Furthermore, the additional components present in the BBE, mainly suberin, may hinder the ease of epoxidation. Nevertheless, despite this, the incorporation of the EBBE into cured thermoset networks and the resulting influence on polymer properties based on the amount included were pursued. Proton NMR (Figure S10) confirmed the incorporation of epoxy functional groups.

**Table 2:** Epoxy Equivalent Weight, Number Average Molecular Weight, and Dispersity of Eponex 1510 and EBBE

**epoxy resin**	**EEW** (g/eq)	***M***_**n**_ (g/mol)	***Đ***
Eponex 1510	215 ± 2.4	437 ± 1.4	1.09 ± 0.004
EBBE	523 ± 52	530 ± 1.4	1.54 ± 0.01

The feasibility of utilizing EBBE in commercial epoxy resin systems with alternative curing agents was evaluated. Each sample is seen to exhibit extents of cure >99% (Table 3), supported by FTIR analysis, indicating near-optimal conversion of epoxy functionalities (see Figures S11–S19). Gel content measurements, determined by toluene reflux extraction, further confirmed the formation of highly cross-linked, insoluble networks. However, a decrease in gel content with increasing EBBE content suggests the presence of oligomeric species within the BBE, which reduces the cross-link density of the network, consistent with the decrease in cross-link density found using DMA, also shown in Table 3.

**Table 3:** Extent of Cure, Gel Content, and Cross-Link Density from DMA of EBBE:Eponex 1510 Epoxy Thermosets Cured with Different Amines

**amine curing agent**	**EBBE:Eponex 1510**	**extent of cure (%)**	**gel content (%)**	***v*** (mol/m^3^)
EPIKURE W	0:100	99.0	99	881 ± 87
	25:75	99.9	94	553 ± 268
	50:50	99.9	82	252 ± 121
NC-558	0:100	99.9	98	824 ± 136
	25:75	99.9	93	562 ± 73
	50:50	99.9	89	546 ± 80
DFDA	0:100	99.9	99	967 ± 96
	25:75	99.9	98	546 ± 80
	50:50	99.9	90	186 ± 2

The cured epoxy resins that contain EPIKURE W as the curing agent exhibited decreasing *T*_g_ with increasing EBBE concentration. Alternatively, the cured epoxy resins that contain either NC-558 or DFDA exhibited a general trend of increasing *T*_g_ with increasing EBBE concentration, as seen in Figure 3, with pertinent data presented in Table 4. Such behavior suggests that these cured resins may possess enhanced physical cross-links between the EBBE and biobased curing agents in the glass transition region, despite the trend of decreasing cross-link density with increasing EBBE exhibited by all cured resins. Compared to the 100% Eponex 1510 samples, EBBE samples exhibit lower average epoxy functionality, as seen by the higher EEW, yielding fewer reactive sites available for cross-linking. The EBBE also contains the bulky and rigid structure of the triterpenoids, which may introduce steric hindrance and limit the accessibility of reactive groups while curing. These factors lead to a less densely cross-linked network, which is reflected in the thermomechanical behavior. Gel content measurements also support the trend of decreasing cross-link density with increasing EBBE by showing an increase in the soluble fraction with increasing EBBE content, leading to less densely cross-linked networks. Furthermore, the *E*′ at 25 °C for all cured resins fell within the 2.0–2.5 GPa range, demonstrating no significant decrease in resistance to deformation upon the incorporation of EBBE or the utilization of a biobased curing agent.

**Table 4:** FTIR, DSC, DMA, and TGA (N_2_) Results of EBBE:Eponex 1510 Epoxy Thermosets Cured with Different Amines

**amine curing agent**	EBBE: Eponex 1510	***T***_**g**_ **(°C) DSC**	***T***_**g**_ **(°C) tan δ**	***T***_**g**_ **(°C) (*E*″)**	***E*′ at 25 °C**	**IDT (°C)**	Char (%) @ 700 °C
EPIKURE W	0:100	123 ± 1	128 ± 1	121 ± 1	1.9 ± 0.8	334 ± 5	2.2 ± 0.28
	25:75	107 ± 0	115 ± 2	105 ± 3	2.5 ± 0.03	284 ± 2	3.1 ± 0.39
	50:50	100 ± 2	110 ± 1	95 ± 0	2.4 ± 0.2	315 ± 2	3.2 ± 0.48
NC-558	0:100	55 ± 1	71 ± 1	59 ± 1	2.2 ± 0.0	300 ± 2	2.7 ± 2.69
	25:75	67 ± 1	81 ± 1	66 ± 2	2.1 ± 0.5	288 ± 0	4.4 ± 0.37
	50:50	75 ± 3	83 ± 6	64 ± 8	2.8 ± 0.1	282 ± 5	4.6 ± 0.44
DFDA	0:100	78 ± 0	81 ± 3	72 ± 3	2.4 ± 0.3	292 ± 1	13.1 ± 0.56
	25:75	78 ± 2	86 ± 0	77 ± 1	2.8 ± 0.2	284 ± 1	12.2 ± 0.32
	50:50	85 ± 1	92 ± 1	78 ± 1	2.1 ± 0.2	278 ± 1	10.9 ± 0.41

**Figure 3:**
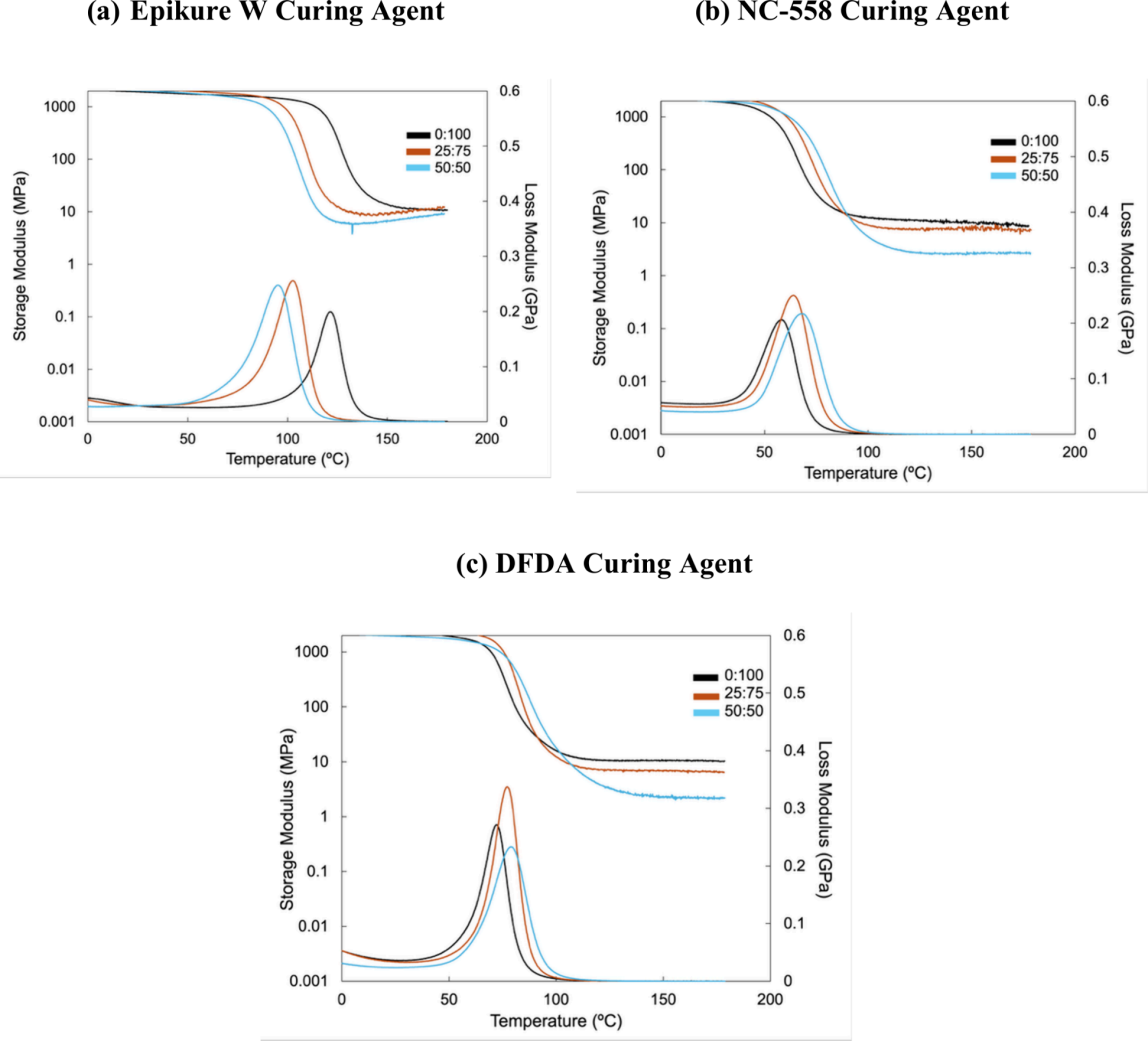
Representative DMA thermograms of the cured epoxy resins.

The thermal behavior of the cured epoxy resins in N_2_ is shown in Figure 4, with pertinent data presented in Table 4. The initial decomposition temperature (*IDT*) (or *T*_95%_) for all the cured resins was measured to be greater than 275 °C. The increased mass loss at lower temperatures for samples with higher EBBE content is attributed to a higher fraction of soluble, lower molecular weight fractions, consistent with gel content analysis, which are more prone to volatilization. The maximum degradation rate for all the cured resins fell within the range of 300–450 °C, while the cured resins containing DFDA exhibited the highest char yields at 700 °C, which has been seen in previous studies that utilized DFDA as the epoxy curing agent.^34,23^

**Figure 4:**
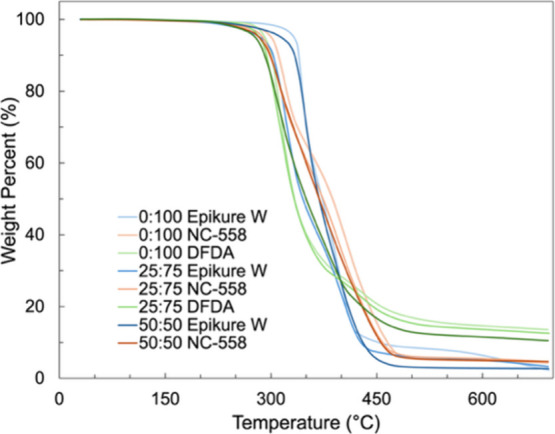
Representative TGA thermograms of the cured epoxy resins in N_2_.

The cured epoxy thermosets exhibited distinct visual differences based on the curing agent, including variations in the color and transparency, as shown in Figure 5. Samples cured with EPIKURE W appeared yellow, while those cured with NC-558 appeared red, and DFDA samples appeared brown. Despite the color differences, all samples had homogeneous characteristics. These differences in color arise from the variations in curing agent structures and their interactions in the epoxy network, which also influence the behavior observed in the DMA results. Images of each ratio of EBBE:Eponex 1510 and curing agents are shown in the SI (see Figure S23).

**Figure 5:**
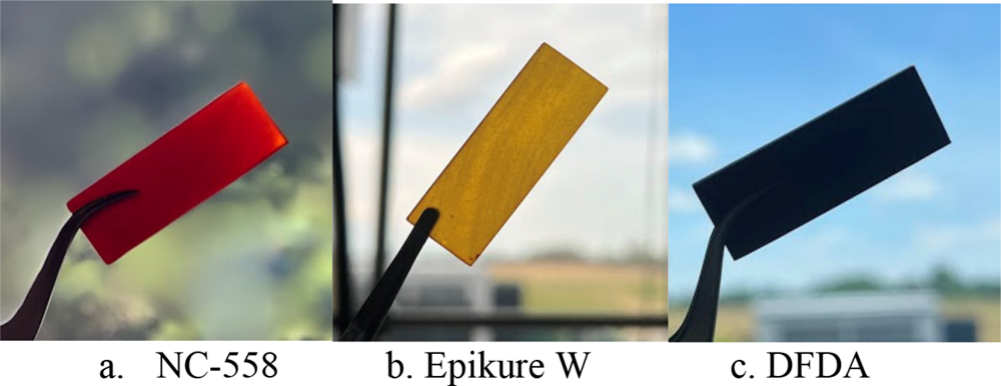
Photographs of EBBE:Eponex 1510 epoxy thermosets with (a) NC-558, (b) EPIKURE W, and (c) DFDA, showing variations in color depending on the curing agent.

## 4 Conclusions

Overall, this work demonstrates that minimally processed BBE can serve as a viable and practical renewable feedstock for epoxy thermoset synthesis without the need for costly or extensive purification steps. Rather than relying on isolated products, this approach allows for the natural chemical complexity of biocrude extracts to be used in the development of thermoset polymers with promising thermomechanical and thermal properties. Although chloroform was used as an extraction solvent, future work will investigate using ethanol-extracted BBE as the starting material to improve the environmental sustainability of this approach.

EBBE was successfully synthesized through an epoxidation of BBE with EpCl, followed by purification steps. The limitation of using EBBE is that it is solid at room temperature, but the addition of Eponex 1510 allowed the EBBE to easily mix and be poured into molds. The EBBE blends were successfully cured with three different amines, EPIKURE W, NC-558, and DFDA, to investigate the thermomechanical and thermal performance of the final cured epoxy resins. The incorporation of EBBE did decrease the *T*_g_ of the epoxy resins cured with EPIKURE W, but it did not alter the *T*_g_ values of the epoxy resins cured with biobased curing agents, while all cured resins exhibited glassy storage moduli in the range of 2.0–2.5 GPa and thermal stabilities up to 275 °C in N_2_.

The ability to incorporate BBE into commercial epoxy systems helps create a pathway for partial replacement of petroleum-based resins. This strategy, which tolerates the chemical complexity of the feedstocks and minimizes the number of processing steps, is intended to address two major hurdles that have hindered the scaling-up of biobased thermosets. This research also demonstrates that forestry and pulp and paper byproducts should be recognized as valuable materials rather than just fuels and can contribute to a more sustainable way of making high-performance polymers.

## Supplementary Material



## Notes

Representative chemical structure of suberin; photographs of extract, uncured epoxy, and cured epoxy with curing agents; HPLC and APC chromatograms of extract and epoxy; proton NMR spectra of extract and epoxy; FTIR spectra of epoxy before and after curing; and overlay graphs of DSC, tan δ, and loss modulus data (PDF)
