# Supplementary material for: Evaluation of Functionalized Birch Bark Extracts in Epoxy Thermosets
Source: Biomacromolecules. 2026 Jun 24;27(7):4298–305. doi: 10.1021/acs.biomac.6c00228 (PMC13370770; doi:10.1021/acs.biomac.6c00228)
Supplement: Supplementary file 1 [file bm6c00228_si_001.pdf]

Electronic Supporting Information

Evaluation of Functionalized Birch Bark Extracts in Epoxy Thermosets

Heather M. LaFrance[1,2]*, John D. Chea*[1,2]*, Emre Kinaci*[1,2]*, Kylie D. Howard*[1,2]*, James A. Newell*[1,2]*, and Joseph F. Stanzione, III*[1,2*]

[1]Department of Chemical Engineering, Rowan University, 201 Mullica Hill Rd., Glassboro, NJ, 08028, USA

[2]Advanced Materials and Manufacturing Institute (AMMI), Rowan University, 107 Gilbreth Pkwy, Mullica Hill, NJ 08062, USA

*Corresponding Author: Joseph F. Stanzione, III, Professor of Chemical Engineering and Founding Director of AMMI

Email address: stanzione@rowan.edu

Heather LaFrance, Ph.D. student

Email address: lafran12@students.rowan.edu

John D. Chea, Ph.D. student

Email address: cheaj2@rowan.edu

Emre Kinaci, Ph.D., Research Scientist, Assistant Director of AMMI

Email address: kinaci@rowan.edu

Kylie Howard, Ph.D. student

Email address: howard26@rowan.edu

James Newell, Ph.D., Professor of Chemical Engineering

Email address: newell@rowan.edu

Chemical Structures

Figure S1. Representative Structure of Suberin

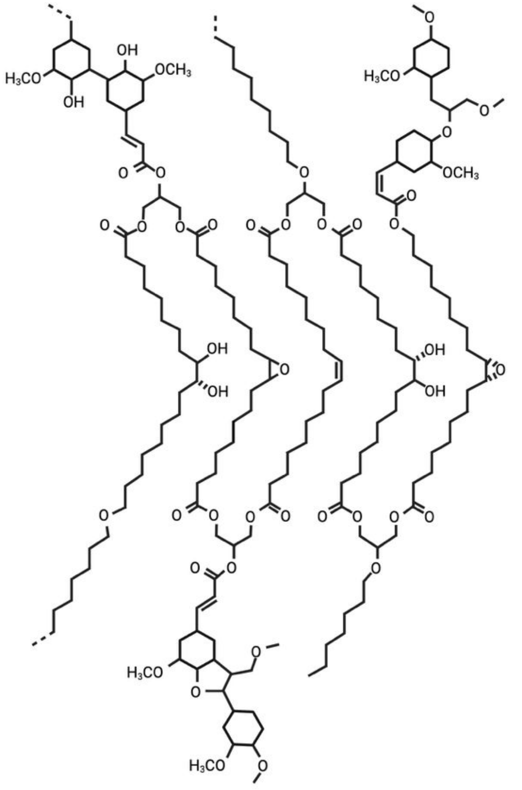

Figure S2. Chloroform BBE

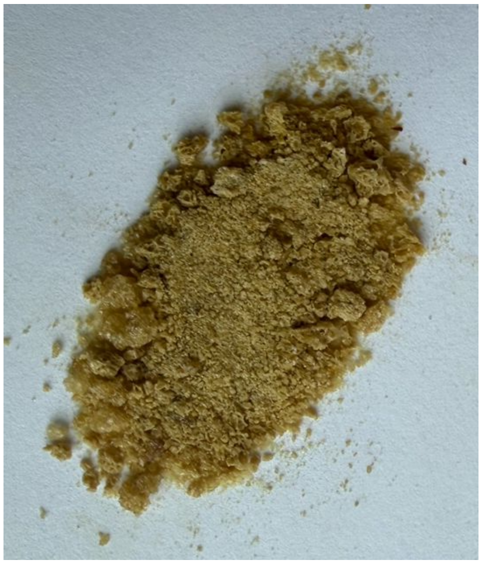

Figure S3. Ethanol BBE

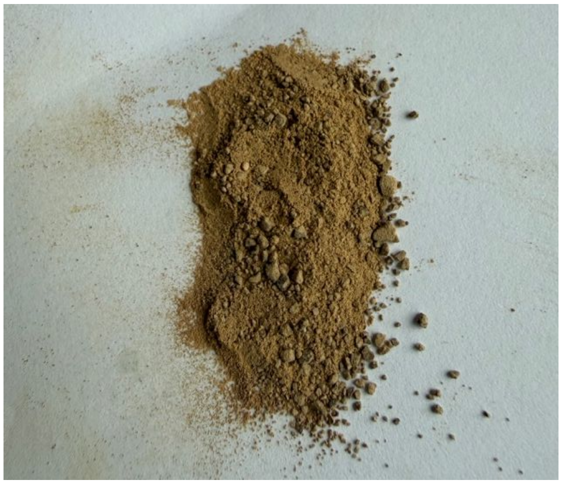

Figure S4. Chloroform EBBE

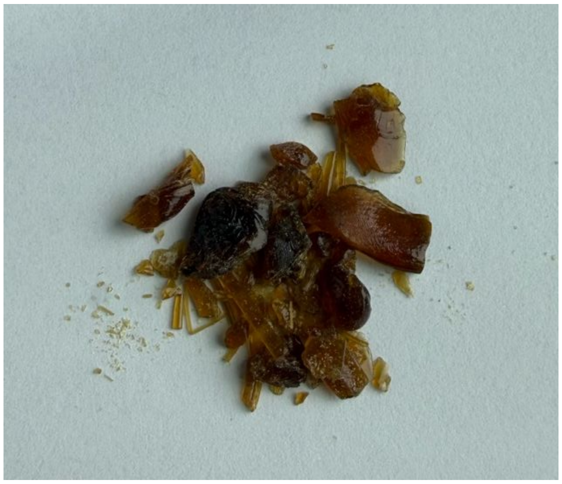

High Performance Liquid Chromatography

Figure S5. Chloroform BBE

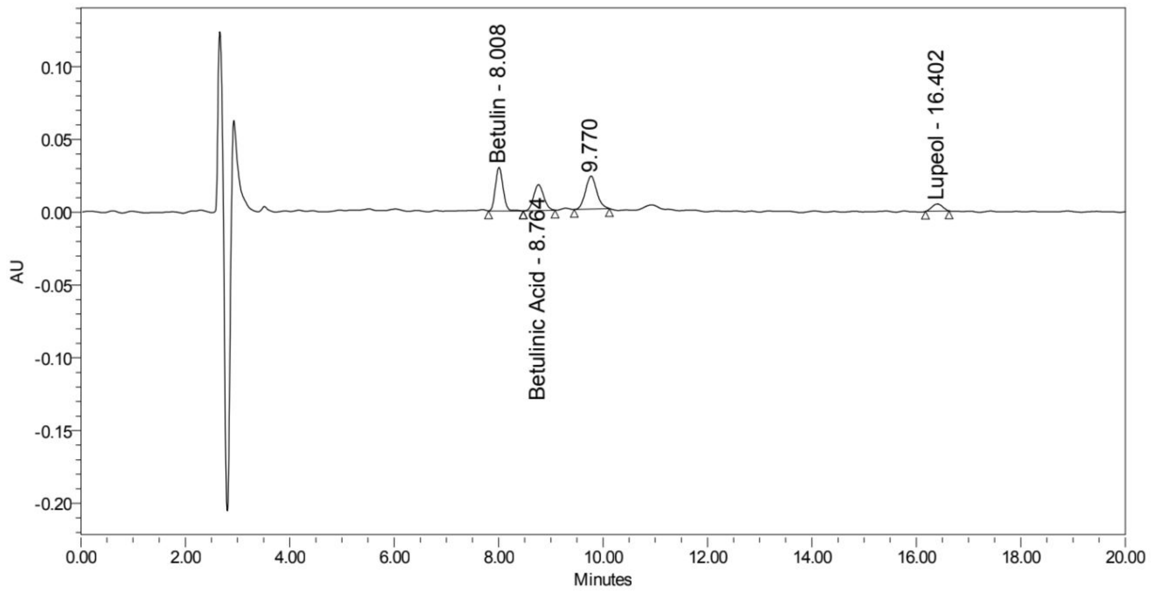

Figure S6. Ethanol BBE

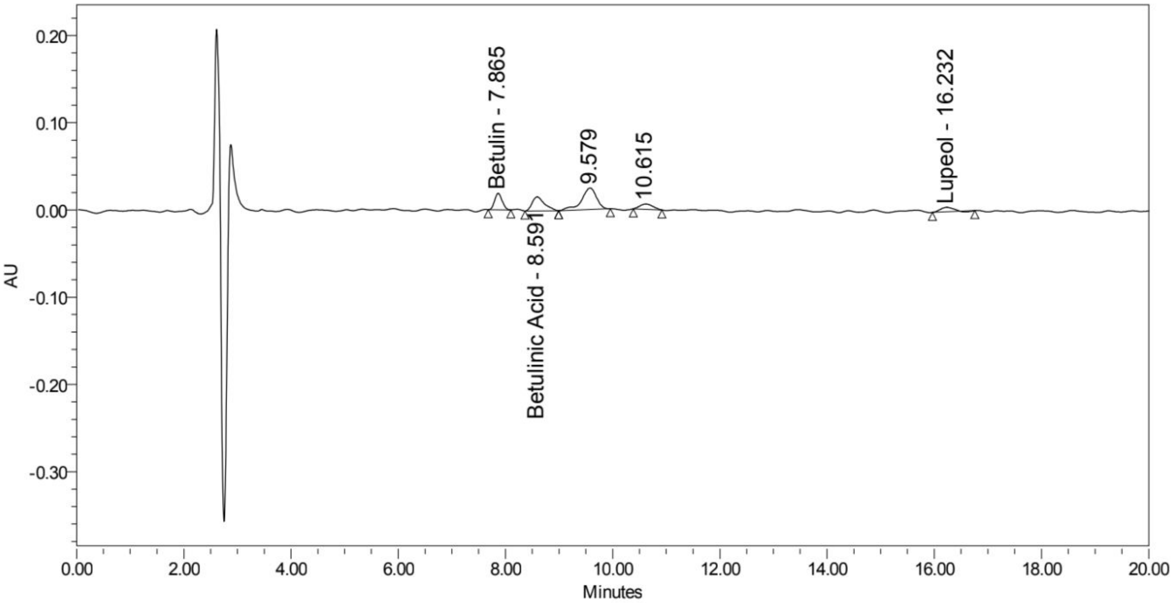

Advanced Polymer Chromatography (APC)

Figure S7. Chloroform BBE

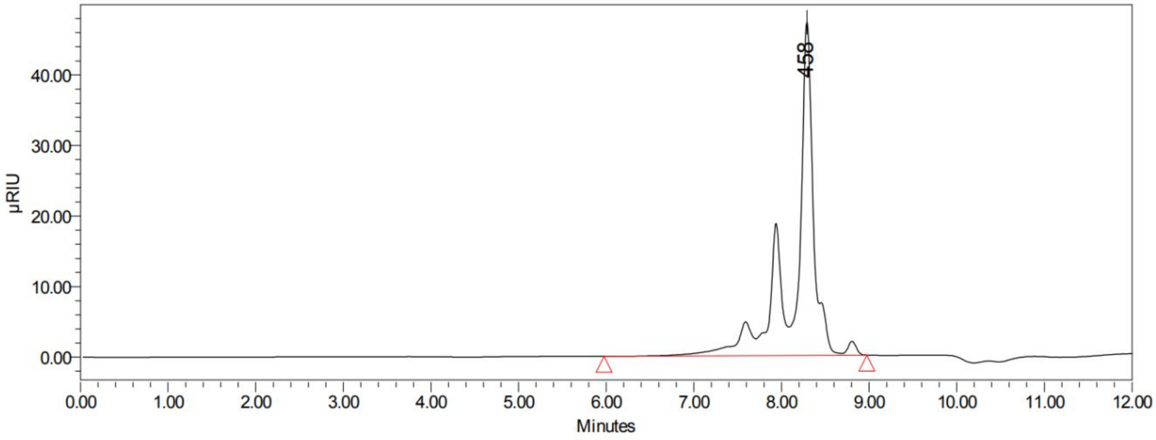

Figure S8. Ethanol BBE

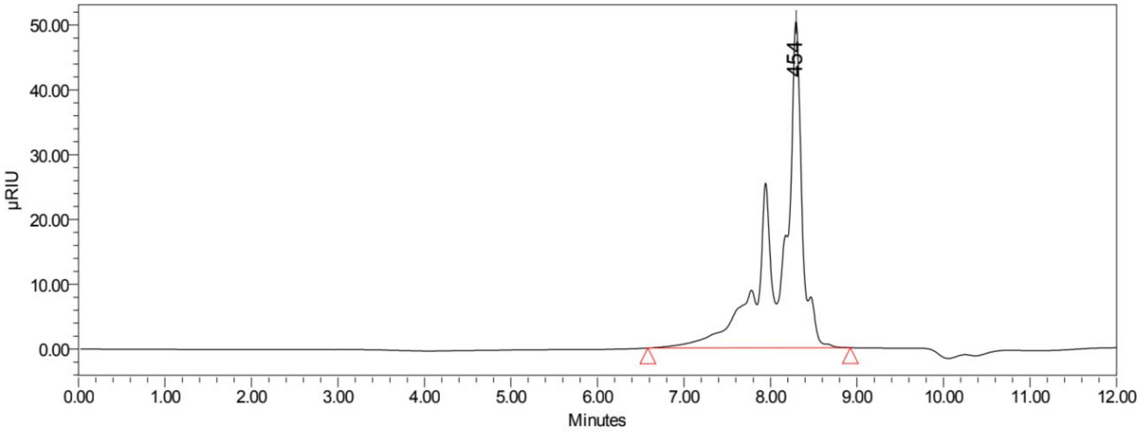

Figure S9. Chloroform EBBE

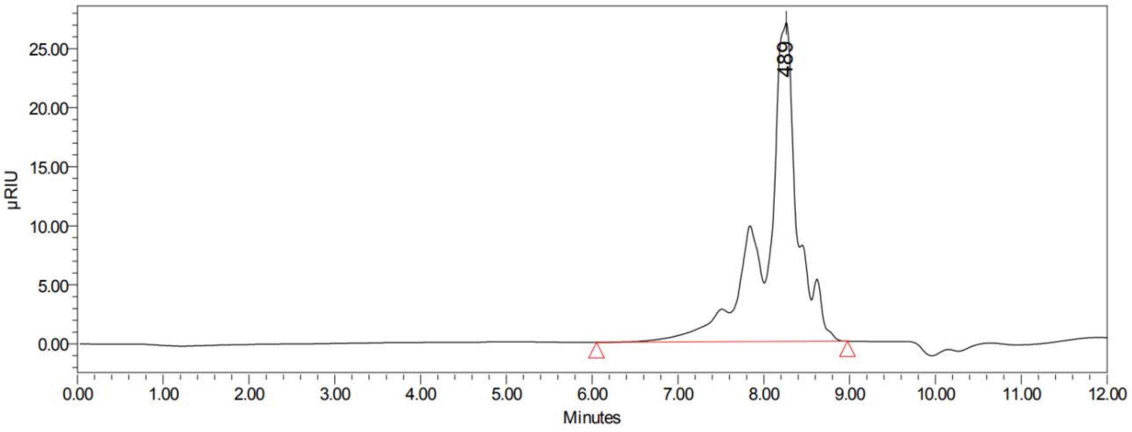

Nuclear Magnetic Resonance (NMR)

Figure S10. BBE vs EBBE Proton NMR Data

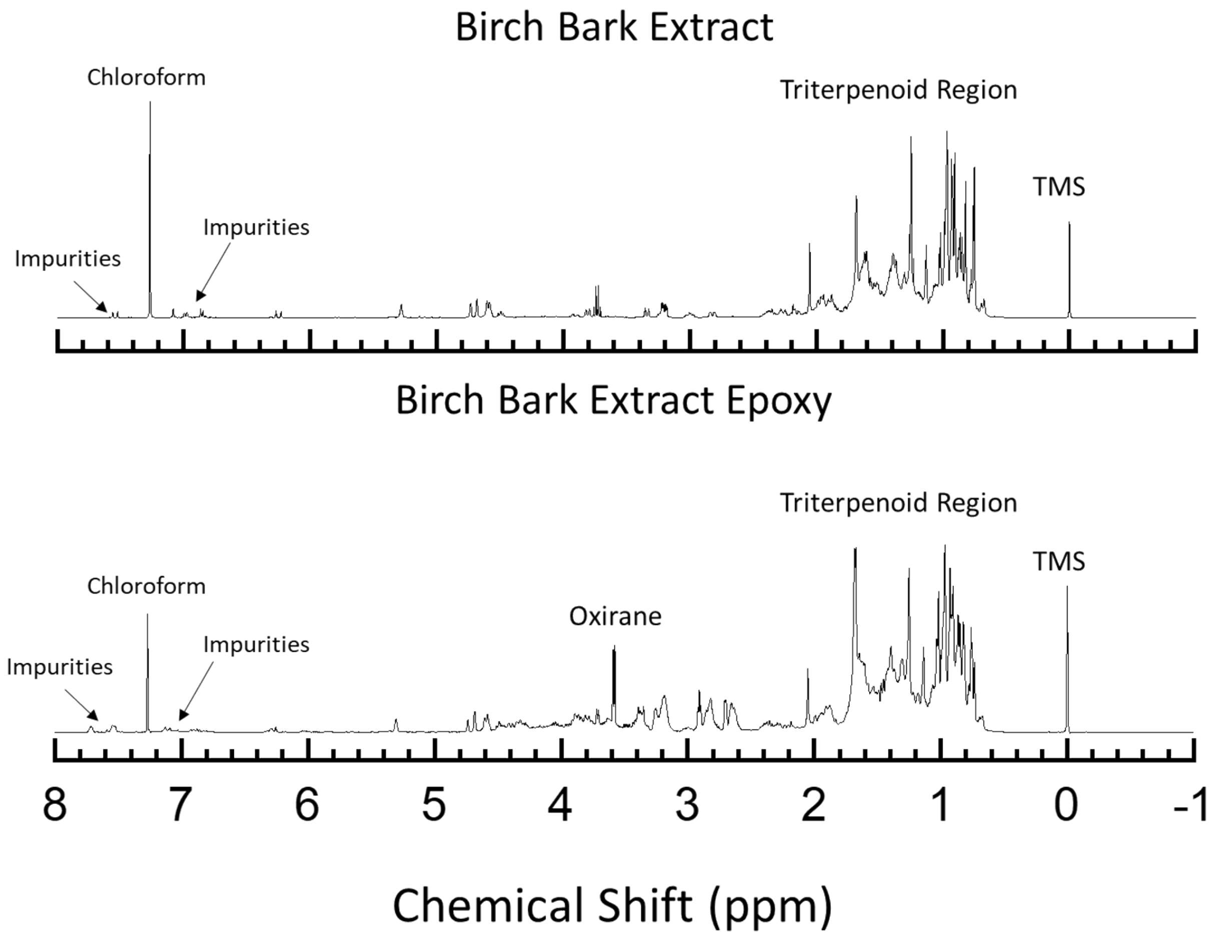

Fourier Transform Infrared Spectroscopy (FTIR)

Figure S11. FTIR comparison plots of Eponex 1510 cured with Epikure W

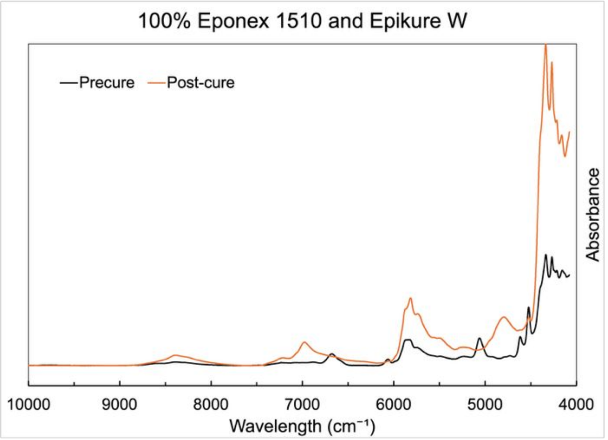

Figure S12. FTIR comparison plots of Eponex 1510 cured with NC-558

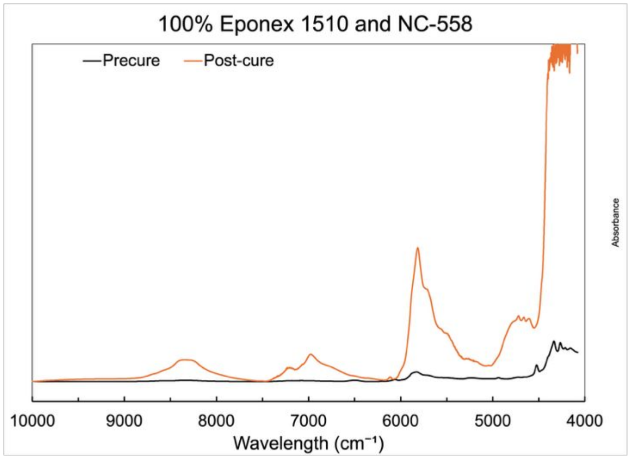

Figure S13. FTIR comparison plots of Eponex 1510 cured with DFDA

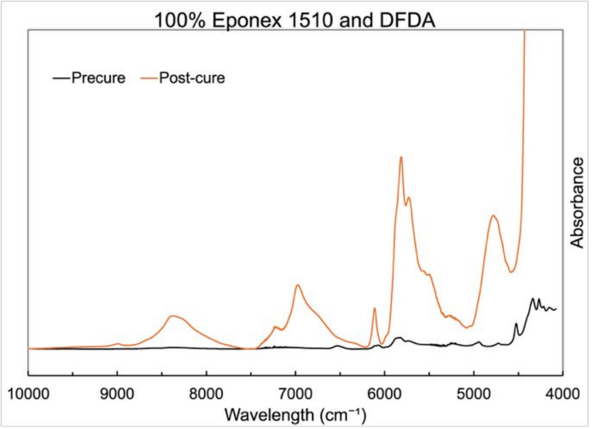

Figure S14. FTIR comparison plots of 25:75 EBBE:Eponex 1510 cured with Epikure W

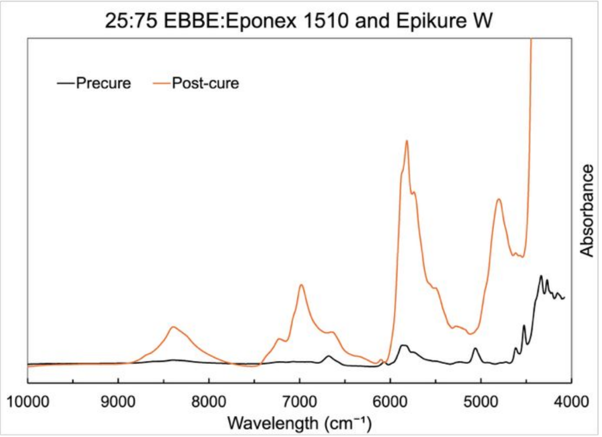

Figure S15. FTIR comparison plots of 25:75 EBBE:Eponex 1510 cured with NC-558

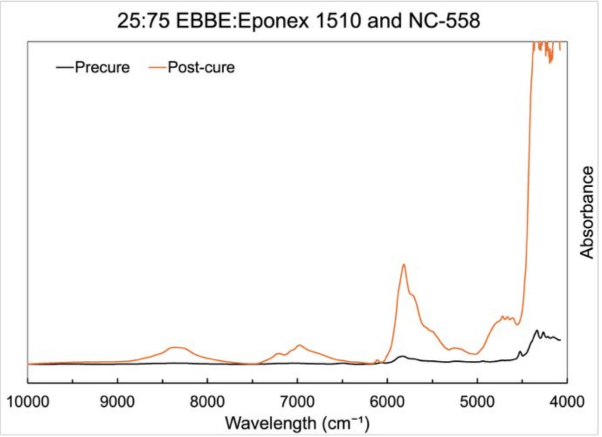

Figure S16. FTIR comparison plots of 25:75 EBBE:Eponex 1510 cured with DFDA

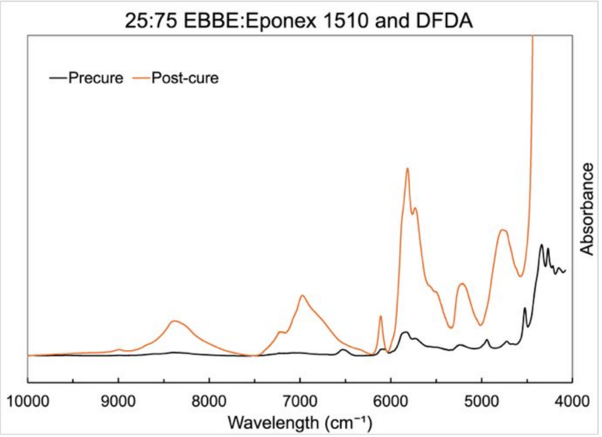

Figure S17. FTIR comparison plots of 50:50 EBBE:Eponex 1510 cured with Epikure W

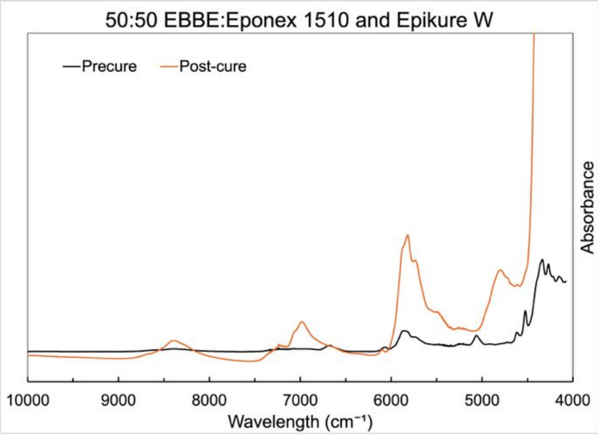

Figure S18. FTIR comparison plots of 50:50 EBBE:Eponex 1510 cured with NC-558

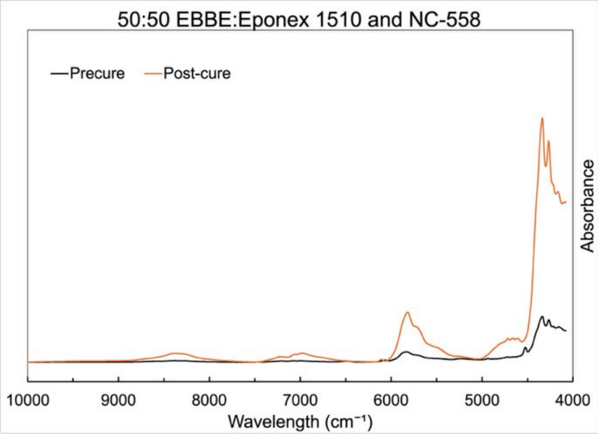

Figure S19. FTIR comparison plots of 50:50 EBBE:Eponex 1510 cured with DFDA

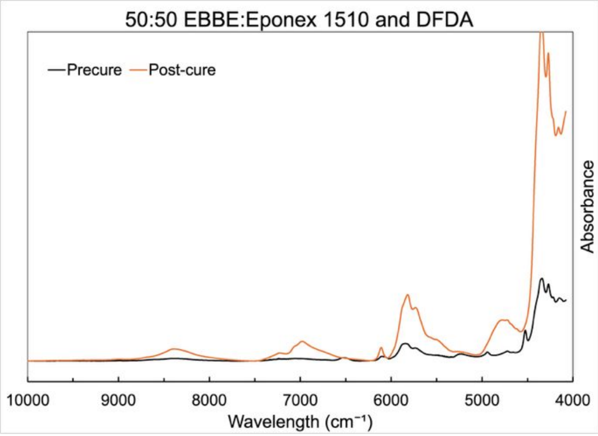

Glass Transition Temperature from DSC and DMA

Figure S20. Samples cured with Epikure W

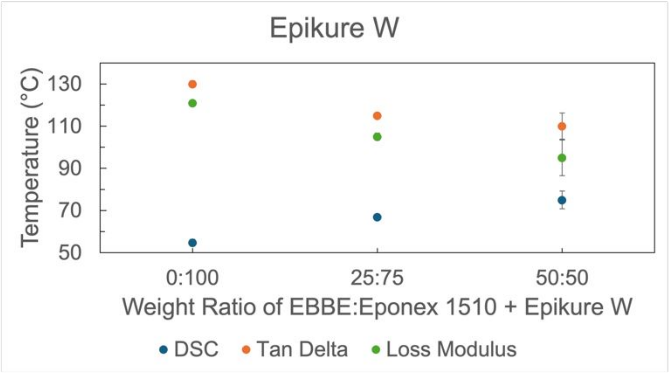

Figure S21. Samples cured with NC-558

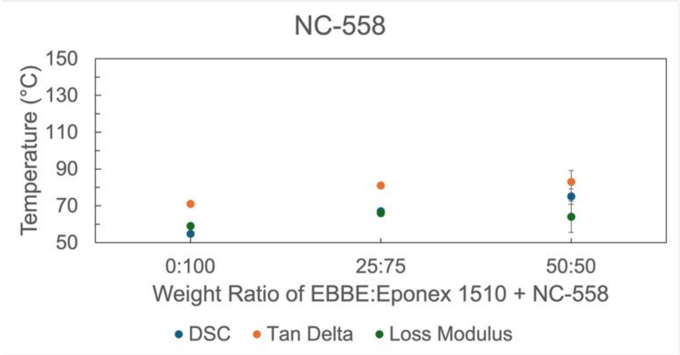

Figure S22. Samples cured with DFDA

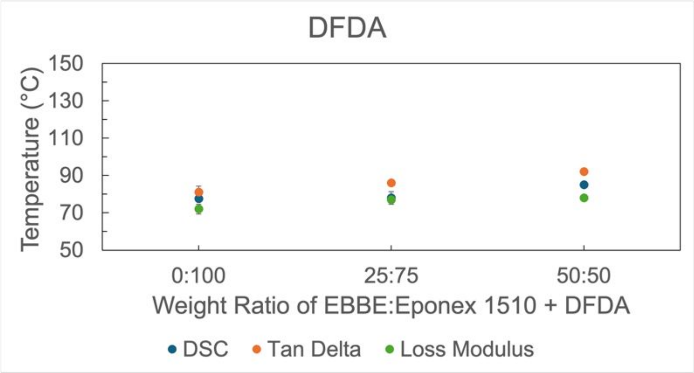

Figure S23. Cured Thermosets with Different Amines

0:100 EBBE:Eponex 1510 + Epikure W	0:100 EBBE:Eponex 1510 + NC-558	0:100 EBBE:Eponex 1510 + DFDA
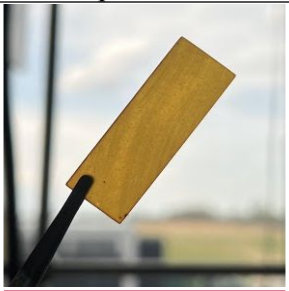	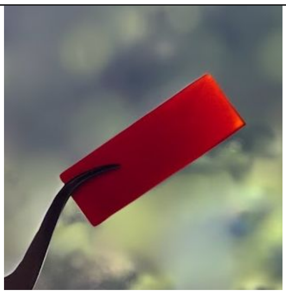	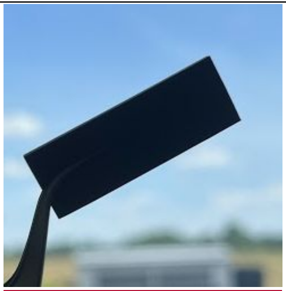
25:75 EBBE:Eponex 1510 + Epikure W	25:75 EBBE:Eponex 1510 + NC-558	25:75 EBBE:Eponex 1510 + DFDA
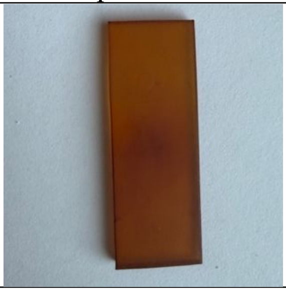	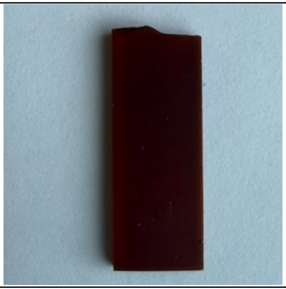	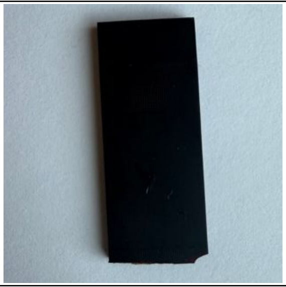
50:50 EBBE:Eponex 1510 + Epikure W	50:50 EBBE:Eponex 1510 + NC-558	50:50 EBBE:Eponex 1510 + DFDA
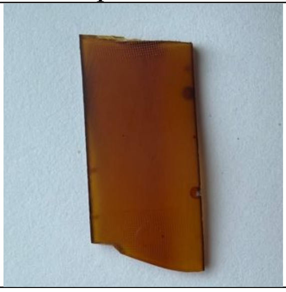	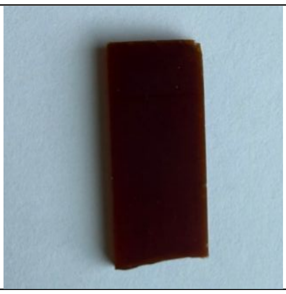	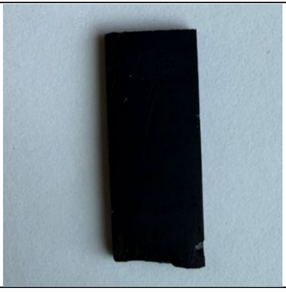
